# An Explanation about Premiums

**Published:** 1896-02

**Authors:** 


### AN EXPLANATION ABOUT PREMIUMS.

Having lately received orders from some old subscribers for "Stories of a Country Doctor," without the full payment of a *full year's subscription in advance*, we deem it necessary to again say that we only send this premium to those who pay *at least a full year in advance.*

Paying for a year nearly ended, or paying dues for which you have received a bill does not entitle you to a premium. A full year in advance of back dues must be paid. If we sent the premium to all who pay anything we would soon find it a losing business.

